# Supplementary material for: Simulating the impact of non-pharmaceutical interventions limiting transmission in COVID-19 epidemics using a membrane computing model
Source: Microlife. 2021 Sep 9;2:uqab011. doi: 10.1093/femsml/uqab011 (PMC8499911; doi:10.1093/femsml/uqab011)
Supplement: uqab011_Supplemental_File [file uqab011_supplemental_file.docx]

| **20% reduction in contagions** | | | | | | |
| --- | --- | --- | --- | --- | --- | --- |
|  | **w/o** | **day 45** | **day 37** | **day 30** | **day 23** | **day 15** |
| asymptomatic | 37.43%  (day 43) | 37.43%  (day 43) | 34.12%  (day 43) | 32.89%  (day 44) | 33.50%  (day 44) | 31.58%  (day 48) |
| WS-RW | 3.71%  (day 57) | 3.39%  (day 55) | 3.15%  (day 57) | 2.94%  (day 62) | 3.09%  (day 60) | 3.08%  (day 62) |
| WS-EW | 1.97%  (day 52) | 2.08%  (day 50) | 1.85%  (day 58) | 1.47%  (day 64) | 1.72%  (day 56) | 1.72%  (day 60) |
| SS-EW | 1.36%  (day 56) | 1.15%  (day 62) | 1.33%  (day 66) | 1.10%  (day 61) | 1.39%  (day 62) | 1.10%  (day 65) |
| SS-SW | 0.58%  (day 60) | 0.56%  (day 57) | 0.43%  (day 68) | 0.47%  (day 69) | 0.51%  (day 61) | 0.49%  (day 63) |
| **50% reduction in contagions** | | | | | | |
|  | **w/o** | **day 45** | **day 37** | **day 30** | **day 23** | **day 15** |
| asymptomatic | 37.43%  (day 43) | 37.43%  (day 43) | 29.40%  (day 41) | 24.20%  (day 48) | 21.54%  (day 55) | 19.84%  (day 62) |
| WS-RW | 3.71%  (day 57) | 3.18%  (day 54) | 2.16%  (day 63) | 2.25%  (day 66) | 1.92%  (day 83) | 1.71%  (day 90) |
| WS-EW | 1.97%  (day 52) | 2.01%  (day 50) | 1.10%  (day 47) | 1.19%  (day 68) | 0.97%  (day 83) | 0.84%  (day 91) |
| SS-EW | 1.36%  (day 56) | 1.21%  (day 58) | 0.78%  (day 72) | 0.85%  (day 75) | 0.59%  (day 82) | 0.69%  (day 97) |
| SS-SW | 0.58%  (day 60) | 0.48%  (day 65) | 0.30%  (day 65) | 0.38%  (day 72) | 0.33%  (day 86) | 0.30%  (day 101) |
| **80% reduction in contagions** | | | | | | |
|  | **w/o** | **day 45** | **day 37** | **day 30** | **day 23** | **day 15** |
| asymptomatic | 37.43%  (day 43) | 37.43%  (day 43) | 28.98%  (day 37) | 12.04%  (day 30) | 4.48%  (day 62) | 2.70%  (day 125) |
| WS-RW | 3.71%  (day 57) | 2.95%  (day 48) | 1.55%  (day 40) | 0.72%  (day 42) | 0.31%  (day 45) | 0.16%  (day 135) |
| WS-EW | 1.97%  (day 52) | 2.09%  (day 50) | 1.08%  (day 42) | 0.49%  (day 36) | 0.15%  (day 49) | 0.18%  (day 61) |
| SS-EW | 1.36%  (day 56) | 1.01%  (day 52) | 0.66%  (day 45) | 0.29%  (day 39) | 0.13%  (day 45) | 0.15%  (day 73) |
| SS-SW | 0.58%  (day 60) | 0.36%  (day 53) | 0.23%  (day 47) | 0.11%  (day 59) | 0.07%  (day 51) | 0.06%  (day 67) |

**Table S1.** Detailed values of Figure 2, showing the percentage reduction in maximum prevalence (RMP) in the various COVID-19 immunological response populations (see text of Figure 1) according to the time interventions were adopted and according to the intensity of the reduction in transmission.
